# The Reaction of 4,5-Dichloro-1,2,3-dithiazolium Chloride with Sulfimides: A New Synthesis of *N*-Aryl-1,2,3-dithiazolimines

**DOI:** 10.3390/molecules14072356

**Published:** 2009-07-02

**Authors:** Andreas S. Kalogirou, Panayiotis A. Koutentis

**Affiliations:** Department of Chemistry, University of Cyprus, P.O. Box 20537, 1678 Nicosia, Cyprus; E-mail: andreas_ch05@hotmail.com (A-S.K.)

**Keywords:** dithiazole, dithiazolimine, sulfimide, sulfilimine, heteroarene, appel salt

## Abstract

*N*-Aryl-*S*,*S*-dimethylsulfimides **3** (Ar = **4**-NO_2_C_6_H_4_), **4** (Ar = Ph) and **5** (Ar = 4-Tol) react with Appel salt **1** to give the corresponding *N*-aryl-(4-chloro-5*H*-1,2,3-dithiazolylidene)benzenamines **8** (Ar = 4-NO2C6H4), **9** (Ar = Ph) and **10** (Ar = 4-Tol) in 84, 94 and 87% yields, respectively. The reaction proceeds in the absence of base and a proposed reaction mechanism is given.

## 1. Introduction

*N*-Aryl-1,2,3-dithiazol-5*H*-imines show interesting antitumour [1], antibacterial [2,3,4], antifungal [5,6,7], and herbicidal [8] activities. The biological activity could be due to the 1,2,3-dithiazole ring, which acts as a powerful inhibitor of several enzymes that are structurally related to serine proteases [9]. Furthermore *N*-aryldithiazolimines are useful precursors to other heterocycles through ANRORC [10,11] style ring transformations. For example the thermolysis of *N*-aryldithiazolimines can afford benzothiazoles [12,13], benzimidazoles [14], thiazolopyridines [15] and benzoxazines [16].

**Scheme 1:**
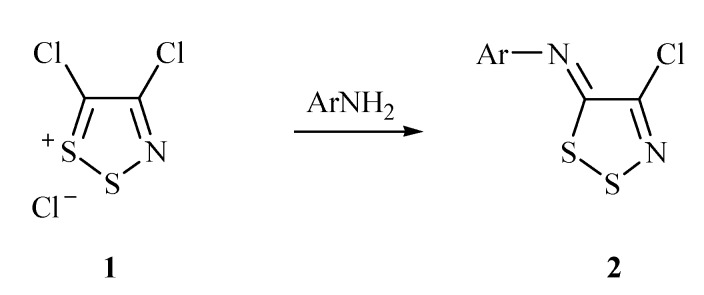
The classical reaction of anilines with Appel salt **1** to afford dithiazolimines **2**.

Most primary arylamines react readily with 4,5-dichloro-1,2,3-dithiazolium chloride **1** (Appel salt) [9,17,18,19] to give, after treatment with tertiary amine base (2 equiv.), the corresponding *N*-aryl-4-chloro-5*H*-1,2,3-dithiazolimines **2** in good to excellent yields [20,21] (Scheme 1). In some cases, such as with arylamides [22], heteroarylamines [21,23] or alkylamines [20,21], the reactions are low yielding or complex. As such this simple condensation reaction has room for improvement.

Sulfimides act as transfer reagents in the form of an “activated amine”. For example sulfimides react with nitrile oxides to afford 1*H*-l,2,4-triazole 2-oxides [24], and react with alkoxychromium (Fischer) carbenes to form imidates [25]. In view of their use as *N*-transfer reagents to electrophiles, we examined an alternative route to *N*-aryl-1,2,3-dithiazolimines by reacting *N*-aryl-*S*,*S*-dimethyl-sulfimides with Appel salt **1**.

## 2. Results and Discussion

We were able to prepare five sulfimides according to literature procedures (**3**, R = 4-NO_2_C_6_H_4_ [26]; **4**, R = Ph [26]; **5**, R = 4-Tol [26]; **6**, R = Pyrid-2-yl [27]; and **7**, R = Bz [26]). Disappointingly, treating Appel salt **1** with either the *N*-pyrid-2-yl or *N*-benzoyl sulfimides **6** and **7** (1 equiv.) in DCM (dry) at *ca.* 20 ^o^C gave only complex reaction mixtures (by TLC) that were not investigated further. Nevertheless the three *N*-aryl-sulfimides **3**-**5** reacted rapidly with Appel salt **1** to give the anticipated *N*-aryl-(4-chloro-5*H*-1,2,3-dithiazol-5-imines) **8-10** in excellent yields (84, 94 and 87%, respectively), comparable to those obtained in our hands from the classical [21] condensation of Appel salt **1** with the corresponding aniline (1 equiv.) and pyridine (2 equiv.) (Table 1).

**Table 1:** Reaction of Appel salt **1** (0.96 mmol) with: sulfimides (Method A) and anilines (Method B), in dry DCM, at *ca.* 20 °C.

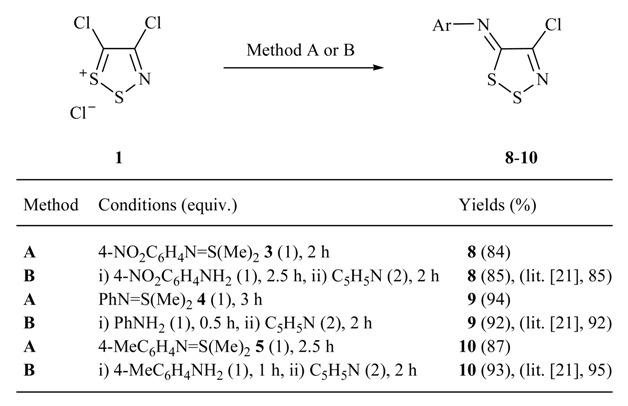

**Scheme 2:**
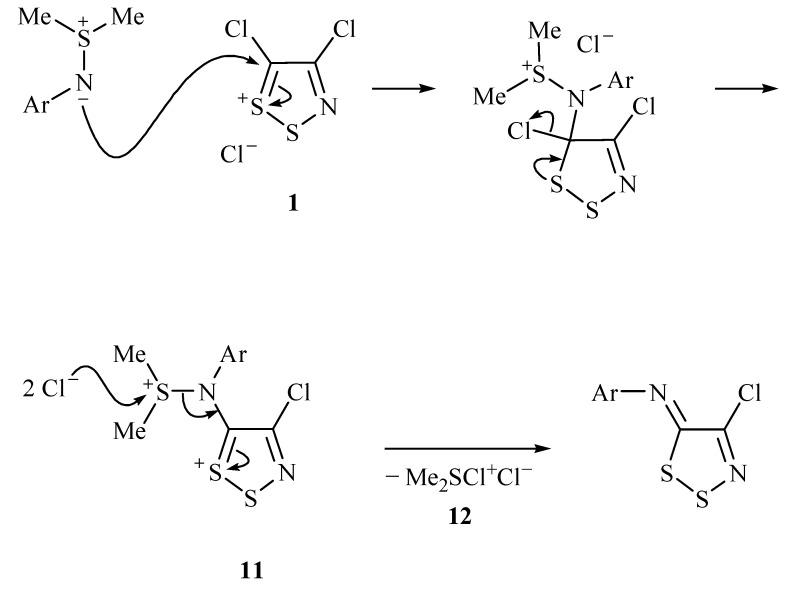
Proposed reaction mechanism for the reaction of sulfimide with 4,5-dichloro-1,2,3-dithiazolium chloride **1**.

Repeating the reaction of the *N*-(4-nitrophenyl)sulfimide **3** with Appel salt **1** in dry MeCN at *ca.* 20 ^o^C gave marginally lower yields of the dithiazolimine **8** (79%). A tentative mechanism for these reactions is proposed (Scheme 2).

The *N*-aryl-*S*,*S*-dimethylsulfimide can attack Appel salt **1** at the highly electrophilic C-5 position to afford, after elimination of chloride, a new dithiazolium intermediate **11** (Scheme 2). The cationic dimethylsulfonium can depart assisted by chloride or an equivalent species. The proposed chlorodimethylsulfonium chloride **12** byproduct was a well known species and under the reaction conditions can convert into a number of alternative species including DMSO on hydrolysis [28] or dimethylsulfide [29,30] on reductive dechlorination.

It is worth noting however, that while the reaction of Appel salt **1** with *N*-aryl-*S*,*S*-dimethylsulfimides provides an alternative, mild and fast route to dithiazolimines in the absence of base, it has drawbacks owing to the limited availability of a wide range of sulfimide reagents [26,27,31].

## 3. Conclusions

*N*-Aryl-*S*,*S*-dimethylsulfimides **3** (Ar = 4-NO_2_C_6_H_4_), **4** (Ar = Ph) and **5** (Ar = 4-Tol) react with Appel salt **1** to give the corresponding *N*-aryl-(4-chloro-5*H*-1,2,3-dithiazolylidene)benzenamines **8** (Ar = 4-NO_2_C_6_H_4_), **9** (Ar = Ph) and **10** (Ar = 4-Tol) in 84, 94 and 87% yields, respectively. The reaction demonstrates an alternative and mild route to 1,2,3-dithiazolimines which does not require the addition of base (2 equiv.), but it is synthetically limited owing to the poor availability and stability of the required sulfimide reagents.

## 4. Experimental

### 4.1. General

Solvents DCM and MeCN were freshly distilled from CaH_2_ under argon. Reactions were protected from atmospheric moisture by CaCl_2_ drying tubes. Anhydrous Na_2_SO_4_ was used for drying organic extracts, and all volatiles were removed under reduced pressure. All reaction mixtures and column eluents were monitored by TLC using commercial glass backed thin layer chromatography (TLC) plates (Merck Kieselgel 60 F_254_). The plates were observed under UV light at 254 and 365 nm. The technique of dry flash chromatography was used throughout for all non-TLC scale chromatographic separations using Merck Silica Gel 60 (less than 0.063 mm). Melting points were determined using a PolyTherm-A, Wagner & Munz, Koefler-Hotstage Microscope apparatus. Solvents used for recrystallization are indicated after the melting point. UV spectra were obtained using a Perkin-Elmer Lambda-25 UV/vis spectrophotometer and inflections are identified by the abbreviation “inf”. IR spectra were recorded on a Shimadzu FTIR-NIR Prestige-21 spectrometer with a Pike *Miracle* Ge ATR accessory and strong, medium and weak peaks are represented by s, m and w, respectively. ^1^H- and ^13^C-NMR spectra were recorded on a Bruker Avance 300 machine (at 300 and 75 MHz, respectively). Deuterated solvents were used for homonuclear lock and the signals are referenced to the deuterated solvent peaks. Low resolution (EI) mass spectra were recorded on a Shimadzu Q2010 GCMS with direct inlet probe. 4,5-Dichloro-1,2,3-dithiazolium chloride **1** [20], *S*,*S*-dimethyl-*N*-(4-nitrophenyl)-sulfimide **3** [26], *S*,*S*-dimethyl*-N*-phenylsulfimide **4** [26], *S*,*S*-dimethyl-*N*-(4-tolyl)sulfimide **5** [26], *S*,*S*-dimethyl*-N*-(pyrid-2-yl)sulfimide **6** [27], and*N*-benzoyl-*S*,*S*-dimethylsulfimide **7** [26], were prepared according to literature procedures.

### 4.2. Reactions of Appel salt **1** with sulfimides: Typical procedure (see Table 1)

To a stirred solution of 4,5-dichloro-1,2,3-dithiazolium chloride **1** (100 mg, 0.48 mmol) in dry DCM (10 ml) at *ca*. 20 ^o^C, *S*,*S*-dimethyl-*N*-(4-nitrophenyl)sulfimide **3** (95.5 mg, 0.48 mmol) was added in one portion. After 2 h no 4,5-dichloro-1,2,3-dithiazolium chloride remained. The reaction mixture was adsorbed onto silica and chromatography (hexane–DCM, 1 : 1) gave *N*-(4-chloro-5*H*-1,2,3-dithiazol-5-ylidene)-4-nitrobenzenamine **8** (110.1 mg, 84%) as yellow needles, mp 161-162 ^o^C (lit. [5], 160 ^o^C) (from cyclohexane) identical with an authentic sample.

*N-(4-Chloro-5H-1,2,3-dithiazol-5-ylidene)benzenamine* **9**: Similarly treatment of 4,5-dichloro-1,2,3-dithiazolium chloride **1** (100 mg, 0.48 mmol) with *S*,*S*-dimethyl-*N*-phenylsulfimide **4** (73.4 mg, 0.48 mmol) gave the title compound **9** (103.1 mg, 94%) as yellow needles, mp 61-62 ^o^C (lit. [5], 63-65 ^o^C) (from cyclohexane) identical with an authentic sample.

*N-(4-Chloro-5H-1,2,3-dithiazol-5-ylidene)-4-methylbenzenamine* **10**: Similarly treatment of compound **1** (100 mg, 0.48 mmol) with *S*,*S*-dimethyl-*N*-(4-tolyl)sulfimide **5** (80.1 mg, 0.48 mmol) gave the title compound **9** (101.9 mg, 87%) as yellow needles, mp 64-65 ^o^C (lit. [5], 66-67 ^o^C) (from cyclohexane) identical with an authentic sample.

### 4.3. Reactions of Appel salt **1** with anilines: Typical procedure [21] (see Table 1)

To a stirred solution of 4-nitroaniline (66.2 mg, 0.48 mmol) in DCM (2 ml) at *ca*. 20 ^o^C, 4,5-dichloro-1,2,3-dithiazolium chloride **1** (100 mg, 0.48 mmol) was added in one portion. After 2 h no Appel salt **1 **remained and pyridine (80 *μ*l, 0.96 mmol) was added. The mixture was stirred for additional 2 h and then adsorbed onto silica. Chromatography (light petroleum–DCM, 1 : 1) gave *N*-(4-chloro-5*H*-1,2,3-dithiazol-5-ylidene)-4-nitrobenzenamine **8** (110.1 mg, 84%) as yellow needles, mp 161-162 ^o^C (lit. [5], 160 ^o^C) (from cyclohexane) identical to an authentic sample.

*N-(4-Chloro-5H-1,2,3-dithiazol-5-ylidene)benzenamine* **9**: Similarly treatment of aniline (43.8 *μ*l, 0.48 mmol) with 4,5-dichloro-1,2,3-dithiazolium chloride **1 **(100 mg, 0.48 mmol) gave the title compound **9** (100.9 mg, 92%) as yellow needles, mp 61-62 ^o^C (lit. [5], 63-65 ^o^C) (from cyclohexane) identical to an authentic sample.

*N-(4-Chloro-5H-1,2,3-dithiazol-5-ylidene)-4-methylbenzenamine* **10**: Similarly treatment of 4-methylaniline (51.4 mg, 0.48 mmol) with 4,5-dichloro-1,2,3-dithiazolium chloride **1** (100 mg, 0.48 mmol) gave the title compound **10** (111.3 mg, 95%) as yellow needles, mp 64-65 ^o^C (lit. [5], 66-67 ^o^C) (from cyclohexane) identical to an authentic sample.
